# Age trend and risk factors of height loss in Chinese over 40 years old

**DOI:** 10.3389/fendo.2025.1542962

**Published:** 2025-09-08

**Authors:** Yong-Fang Li, Lu-Lu Xu, Qin-Yi Wang, Chuo Luo, Chun Yue, Hong Liu, Zhi-Feng Sheng

**Affiliations:** ^1^ Department of Metabolism and Endocrinology, Zhuzhou Hospital of Xiangya School of Medicine, Central South University, Zhuzhou, China; ^2^ Health Management Center, Hunan Provincial Clinical Medicine Research Center for Intelligent Management of Chronic Disease, The Second Xiangya Hospital of Central South University, Changsha, China; ^3^ Health Management Center, National Clinical Research Center for Metabolic Diseases, Hunan Provincial Clinical Medicine Research Center for Intelligent Management of Chronic Disease, Hunan Provincial Key Laboratory of Metabolic Bone Diseases, Department of Metabolism and Endocrinology, The Second Xiangya Hospital of Central South University, Changsha, China

**Keywords:** height loss, risk factors, bone mineral density, biomarkers, Chinese

## Abstract

**Introduction:**

To evaluate age-related height loss trend in men and women in China, and analyze associated risk factors, we conducted a cross-sectional study among 1286 participants from the Bone Health Improvement Program.

**Methods:**

Laboratory data were derived from the medical system and clinical risk factors of osteoporosis and fracture were collected through questionnaires. BMD was measured by DXA. Height loss was calculated as the difference between currently measured height and the highest self-reported height.

**Results:**

The study cohort comprised 706 males and 580 females, aged between 40 and 80 years, categorized into four age groups. The average height loss among men aged 70–80 was 9.5 times greater than men aged 40-50, while for women it was found to be 7.2 higher. Both genders experienced accelerated height loss after age 60; furthermore, women lose their height faster than men across all age groups. Multivariate logistic regression analysis including clinical risk factors, the best predictors of male height loss ≥4 cm were age (OR = 1.182, 95% CI: 1.111-1.258), rheumatoid arthritis (OR = 0.857, 95% CI: 0.788-0.933) and type 2 diabetes (OR = 3.012, 95% CI: 1.193-7.604), while for women, it was age (OR = 1.278, 95% CI: 1.182-1.382) and height (OR = 0.802, 95% CI: 0.714-0.901). In the multivariate logistic regression analysis involving laboratory data, the best predictive factors for male height loss ≥4 cm were eosinophil ratio (OR = 1.448, 95% CI: 1.11-1.89), fasting plasma glucose (OR = 0.469, 95% CI: 0.287-0.768), glycated hemoglobin (OR = 5.65, 95% CI: 2.511-12.711), and serum sodium levels (OR = 1.328, 95% CI: 1.112-1.585), while females had platelets counts (OR = 0.994, 95%CI: 0.989-1.000), glycated hemoglobin (OR = 1.413, 95% CI: 1.090-1.832), blood urea nitrogen levels (OR = 1.416, 95% CI: 1.147-1.747) and serum globulin levels (OR = 1.092, 95% CI: 1.01-1.18). The ROC curve areas for the models were 92.6%, 87.3%, 79%, and 68.1%, respectively.

**Discussion:**

In Chinese aged over 40 years, the rate of height loss significantly accelerates after age 60, regardless of gender. The discovery of risk factors associated with height loss is beneficial for the early identification of individuals with height loss ≥4 cm, to facilitate the early detection of bone loss and enable further examination and treatment.

## Introduction

Osteoporosis is a systemic skeletal disorder characterized by low bone mass and microarchitectural deterioration of bone tissue, with a consequent increase in bone fragility and susceptibility to fracture (1). It has been termed “the silent epidemic” owing to its long-term, clinically asymptomatic course and the large number of affected subjects (2). With the aging population in China, the prevalence of osteoporosis is rising rapidly; therefore, it has become an important public health concern (3). Fragility fractures represent the most serious complication associated with osteoporosis, resulting not only in pain and disability but also in increased mortality rates, increased healthcare expenditures, and significant financial burdens (3–5). Consequently, it is important to identify early signs of osteoporosis—prior to the occurrence of the first osteoporotic fracture (6). Patients screened for osteoporosis should undergo careful examination to collect data on risk factors associated with osteoporosis and fractures. One of the most important measures is height assessment (2). The Baltimore Longitudinal Study of Aging, which performed serial height measurements over a period of nine years in a cohort of men and women, documented that height loss begins around the age of 30 and increases with age. From age 30 to 70 years, cumulative height loss averaged approximately 3 cm for men and 5 cm for women, and by age 80 years, this figure rose to 5 cm for men and 8 cm for women (7, 8). Height loss occurring with aging can result from several factors, including narrowing of vertebral discs, vertebral compression fractures, or age-related postural changes (9–12). Height loss could serve as a clinical indicator for the identification of vertebral fractures, which are prevalent, yet asymptomatic and often underdiagnosed in older women (12). Beyond the correlation with vertebral fractures, certain data suggests that even modest height loss is associated with an increased risk of non-vertebral fractures, particularly hip fractures, in both women and men (13–15). Kamimura et al. noted that height loss (HL) of 4 cm or more increased fracture risk by 2.3 times in Japanese women 60 years and older (16). Pluskiewicz et al. reported that HL exceeding 4 cm is related to fracture probability above 50% in postmenopausal women (2). Hannan et al. showed that height loss of two or more inches (about 5 cm) was linked to a 40-54% higher risk of hip fracture in adult men (17). Ensrud et al. demonstrated that community-dwelling older men with height loss of 3 cm had nearly twice the risk of hip fracture and a 1.4-fold increased risk of any clinical fracture compared to those with less than 1 cm loss (18). According to A. L. Mikula et al., a loss of 1 cm in height by a man is more indicative of a vertebral fracture than a similar loss by a woman, despite the higher prevalence of fractures among women on a population level (19). However, there remains a paucity of studies that provide differences in age-related changes in height loss among Chinese men and women. In addition, early identification of height loss risk factors and implementation of preventive therapies may facilitate the early detection of bone disorders and the improvement in height loss. The objective of this study was to assess age-related trends in height loss among healthy Han Chinese men and women and to identify risk factors for height loss.

## Methods

### Study participants

We conducted a cross-sectional study using the datasets of the Health Improvement Program of Bone (HOPE), an ongoing prospective study in which patients who underwent physical examination at the Health Management Center of the Second Xiangya Hospital were invited to participate. The HOPE study has been designed to achieve a sample size of 5000 participants over a ten-year period, and as of May 2024, it has already enrolled more than 3846 participants. Patients are eligible for enrollment in the HOPE study if they 1) are ≥40 years old and 2) undergo DXA for BMD measurement. The exclusion criteria are 1) a history of hip joint replacement or lumbar spine surgery, 2) inability to undergo DXA for any reason, 3) a history of treatment with antiosteoporosis drugs, or 4) a history of malignant tumors.

For the present study, 1,286 healthy postmenopausal women and men aged ≥50 years from the HOPE cohort were recruited. Participants who did not complete surveys, physical examinations, or body density measurements were excluded.

Informed consent was obtained from all patients, and the proposal for this study was approved by the Ethics Committee of the Second Xiangya Hospital, Central South University, Changsha, Hunan, China.

### Health survey examinations and laboratory measurements

The health check-up examination includes a questionnaire, anthropometric measurements and laboratory measurements. The questions in the questionnaire included sex, age, alcohol intake, smoking, prior fracture and parental hip fracture history, glucocorticoid use, rheumatoid arthritis, and secondary osteoporosis. Prior fracture history referred to fractures that occurred naturally or under mild external forces during adulthood. Current use of glucocorticoids was defined as either receiving glucocorticoid therapy or having received the equivalent of prednisone >5 mg/d for more than three months. Secondary osteoporosis was defined as osteoporosis due to underlying diseases, such as type 1 diabetes, adult osteogenesis imperfecta, chronic untreated hyperthyroidism, hypogonadism or early menopause (menopause at age <45 years), chronic malnutrition or malabsorption, and chronic liver disease. Alcohol intake was considered significant if intake was ≥3 units/d.

Trained inspectors measure the human body according to standard protocols, including weight, height, waist circumference, and hip circumference. Body mass index (BMI) is defined as the weight (kg) per square meter height (m^2^), expressed in kg/m^2^. Height loss is calculated as the difference between the currently measured height and the highest self-reported height (20). According to the clinical guidelines (21–23), it is suggested that the height loss from peak height that exceeds 4 cm should be used for spinal imaging, and the height loss is categorized into two groups: height loss < 4 cm and height loss ≥4 cm. DXA BMD measurement standards and precision have also been previously detailed (24).

The following laboratory data were measured during physical examination: white blood cell count, red blood cell count, hemoglobin concentration, platelet count, neutrophil percentage, lymphocyte percentage, eosinophil percentage, basophil percentage, fasting plasma glucose, glycosylated hemoglobin, 25-hydroxyvitamin D, serum calcium, serum potassium, serum sodium, serum chloride, blood urea nitrogen, serum creatinine, blood uric acid, alanine aminotransferase (ALT), aspartate aminotransferase (AST), total bilirubin, direct bilirubin, indirect bilirubin, total serum protein, serum albumin, serum globulin, total cholesterol, triglyceride, high-density lipoprotein cholesterol (HDL-C), low-density lipoprotein cholesterol (LDL-C), and total bile acid.

### Statistical analysis

The data are expressed as the average (± standard deviation) of continuous variables and the percentage of classified variables. We use one-way ANOVA to analyze the baseline characteristics of different age groups. The comparison between the two groups, when the data are normally distributed, is analyzed by Student’s t-test, and the non-normal distribution is compared by Mann-Whitney U-test. The classified data were analyzed by chi-square test or Fisher’s exact test. In order to study the independent related risk factors of height loss, the variables with significant differences in univariate analysis were included in multivariate logistic regression analysis (forward: LR method), which is expressed by odds ratios (ORs) and its 95% confidence intervals (CIs). Model 1 included only clinical features, and Model 2 included only biochemical indicators. The Hosmer-Lemeshow test was used for model fitting. The predictive accuracy of the logistic regression model was evaluated by receiver operating characteristic (ROC). Two-tailed p-value <0.05 is considered statistically significant. All the data were analyzed by SPSS software version 23.0 (IBM Corp., Armonk, NY, USA).

## Results

**Figure 1:**
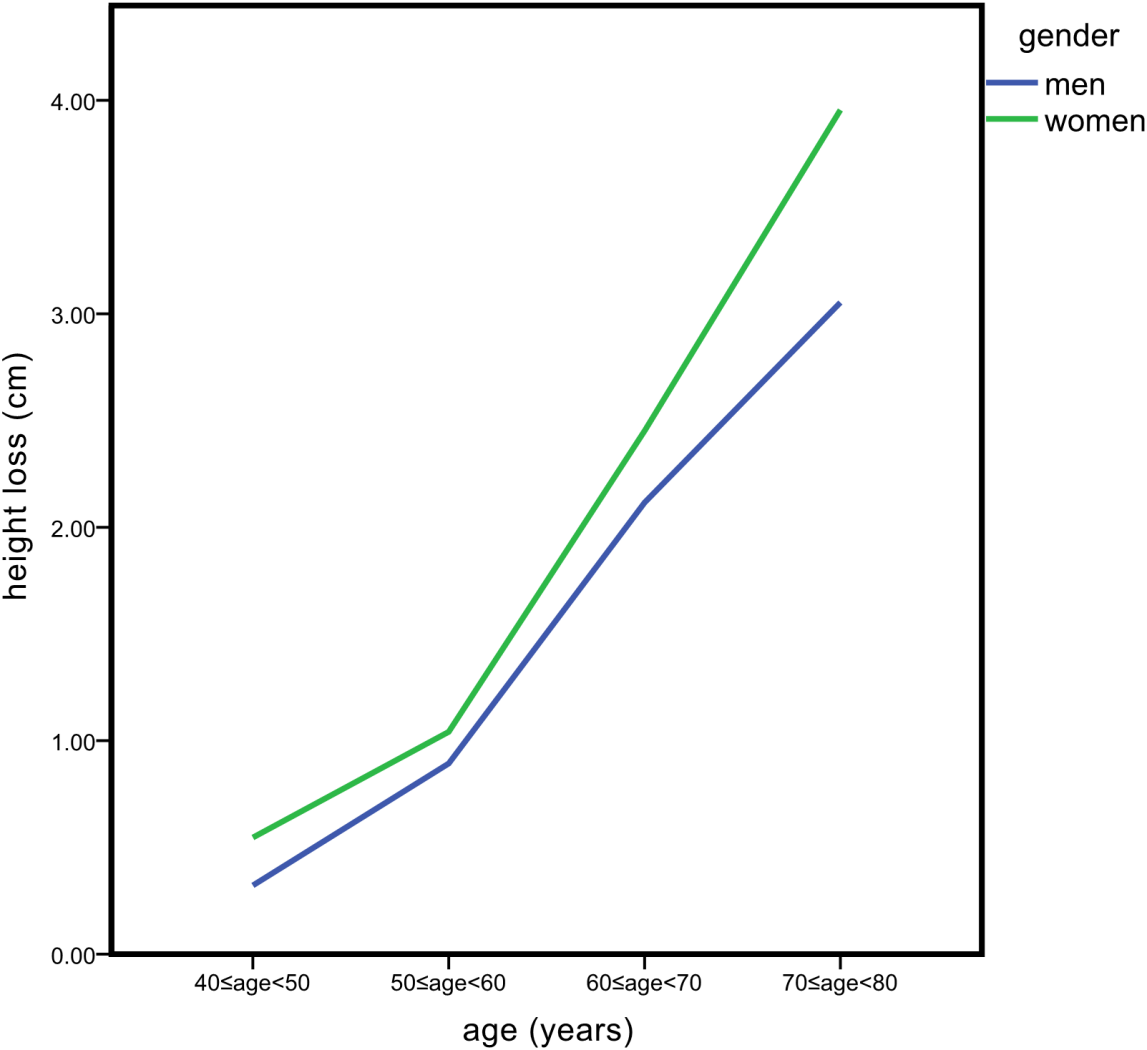
Overall trends of average height loss across age among men and women.

All subjects were divided into 10-year subgroups for cross-sectional analysis by sex. The basic anthropometry and BMD of the 706 men and 580 women are listed in **Tables 1**, **2**, respectively. The highest value of height occurred in the 40–50 age group, and a significant decrease was found at 60 years of age and over in both genders. The loss of height in females was higher than that in males in all age groups. After age 60, the speed of height loss was significantly faster than that before in both genders (**Figure 1**). Height loss in those aged 70–80 years was 9.5 times greater in men and 7.2 times greater in women compared to those aged 40–50 years. Clear declines in BMD with age were evident at the femoral neck and total hip in both sexes. Total lumbar spine BMD decreased significantly with age in women but not in men.

**Table 1:** Basic characteristics of Chinese men.

	Age group (years)				
	40≤age<50 (n=135)	50≤age<60 (n=302)	60≤age<70 (n=190)	70≤age<80 (n=79)	P
Age (years)	45.35±2.71	54.59±2.86	64.52±2.69	73.53±3.1	**<0.001**
Weight (kg)	72.29±9.26	71.56±8.94	68.01±8.66	66±8.38	**<0.001**
Height (cm)	168.91±5.52	168.01±5.42	166.19±5.75	164.1±5.76	**<0.001**
height loss (cm)	0.32±0.75	0.89±1.18	2.12±1.51	3.05±1.97	**<0.001**
BMI (kg/m^2^)	25.31±2.78	25.34±2.86	24.61±2.72	24.49±2.77	**0.007**
waistline (cm)	87.97±7.86	87.85±7.49	87.87±7.72	89.19±7.32	0.612
hipline (cm)	94.95±5.6	93.78±5.49	93.24±5.5	93.01±5.79	**0.041**
Bone mineral densitometry (BMD, g/cm^2^)					
FN-BMD	0.77±0.11	0.76±0.11	0.73±0.1	0.71±0.11	**<0.001**
TH-BMD	0.93±0.12	0.92±0.13	0.9±0.11	0.89±0.12	**0.029**
TL-BMD	0.96±0.12	0.97±0.14	0.96±0.13	0.98±0.15	0.533

BMI, body mass index; FN, femoral neck; TH, total hip; TL, total lumbar.

Significant differences are shown in bold.

**Table 2:** Basic characteristics of Chinese women.

	Age group (years)				
	40≤age<50 (n=86)	50≤age<60 (n=293)	60≤age<70 (n=145)	70≤age<80 (n=56)	P
Age (years)	45.92±2.37	55.11±2.74	64.49±2.85	73.59±2.94	**<0.001**
Weight (kg)	57.79±6.71	57.25±7.35	55.81±7.26	56.37±8.06	0.141
Height (cm)	158.88±4.50	157.43±5.06	154.85±4.86	153.83±5.40	**<0.001**
height loss (cm)	0.55±1.03	1.04±1.33	2.45±1.94	3.96±2.03	**<0.001**
BMI (kg/m^2^)	22.9±2.47	23.09±2.67	23.28±2.88	23.75±2.61	0.26
waistline (cm)	76.22±8.46	78.35±8.19	79.73±7.02	84.23±9.23	**<0.001**
hipline (cm)	92.88±5.6	92.3±5.72	90.53±5.81	92.05±6.27	**0.036**
Bone mineral densitometry (BMD, g/cm^2^)					
FN-BMD	0.75±0.09	0.68±0.09	0.63±0.08	0.57±0.09	**<0.001**
TH-BMD	0.89±0.10	0.82±0.10	0.77±0.10	0.72±0.12	**<0.001**
TL-BMD	0.98±0.11	0.87±0.13	0.8±0.12	0.78±0.12	**<0.001**

BMI, body mass index; FN, femoral neck; TH, total hip; TL, total lumbar.

Significant differences are shown in bold.

The clinical characteristics are listed in **Tables 3**. Among these clinical variables, compared to those with <4 cm of height loss, men with a height loss of ≥4 cm had significant differences in age, weight, height, rheumatoid arthritis, and type 2 diabetes, while women with height loss ≥4 cm had significant differences in age, height, BMI, waistline, prior fracture, rheumatoid arthritis, and type 2 diabetes.

**Table 3:** Clinical characteristics of the study population by category of height loss of Chinese over 40 years old.

	Men			Women		
	height loss <4 cm	height loss ≥4 cm	P	height loss <4 cm	height loss ≥4 cm	P
Number	n=643	n=63		n=504	n=76	
Age (years)	56.71±8.54	67.92±7.80	**<0.001**	56.44±7.32	67.1±8.02	**<0.001**
Weight (kg)	70.46±9.23	65.76±8.95	**<0.001**	57.07±7.13	55.30±8.05	0.052
Height (cm)	167.72±5.58	162.35±5.16	**<0.001**	157.24±4.98	152.21±4.65	**<0.001**
height loss (cm)	1.01±1.13	5.08±1.53	**<0.001**	1.05±1.16	5.29±1.19	**<0.001**
BMI (kg/m^2^)	25.02±2.84	24.94±3.18	0.649	23.07±2.58	23.82±2.93	**0.023**
waistline(cm)	87.88±7.68	88.64±7.84	0.578	78.58±8.09	81.24±8.46	**0.036**
hipline(cm)	93.80±5.60	92.90±5.42	0.128	91.99±5.63	91.22±6.50	0.354
Prior fracture, n (%)	45(7.06%)	7(11.29%)	0.31	54(10.78%)	16(20.78%)	**0.014**
Parental hip fracture, n (%)	70(10.99%)	6(9.68%)	0.686	65(12.97%)	8(10.39%)	0.531
Smoking, n (%)	249(39.09%)	25(9.12%)	0.554	8(1.60%)	0(0.00%)	0.614
Glucocorticoid use, n (%)	11(1.73%)	3(4.84%)	0.121	11(2.20%)	2(2.60%)	0.683
Rheumatoid arthritis, n (%)	10(1.57%)	4(6.45%)	**0.022**	16(3.19%)	9(11.69%)	**0.003**
Alcohol intake, n (%)	145(22.76%)	10(16.13%)	0.232	4(0.80%)	0(0.00%)	1.00
Type 2 Diabetes, n (%)	67(13.62%)	11(39.29%)	**<0.001**	27(6.80%)	11(25.00%)	**0.001**
Secondary osteoporosis, n (%)	49(7.69%)	6(9.68%)	0.342	72(14.37%)	9(11.69%)	0.6
Bone mineral densitometry (g/cm^2^)						
FN-BMD	0.76±0.11	0.7±0.11	**<0.001**	0.68±0.10	0.59±0.10	**<0.001**
TH-BMD	0.92±0.12	0.88±0.12	**0.011**	0.82±0.11	0.73±0.11	**<0.001**
TL-BMD	0.97±0.13	0.96±0.15	0.829	0.88±0.13	0.76±0.13	**<0.001**

BMI, body mass index; FN, femoral neck; TH, total hip; TL, total lumbar.

Significant differences are shown in bold.

The biochemical characteristics are presented in **Tables 4**. Among these biochemical parameters, significant differences were observed in hemoglobin concentration, eosinophil ratio, basophil ratio, fasting plasma glucose, glycosylated hemoglobin, serum sodium levels, and serum albumin levels among men with height loss ≥4 cm compared to those with height loss <4 cm. Similarly, significant differences were found in platelet count, glycosylated hemoglobin, blood urea nitrogen (BUN) levels, serum albumin levels, and serum globulin levels among women with height loss ≥4 cm.

**Table 4:** Biochemical and hematological variables associated with height loss of Chinese over 40 years old.

	Men			Women		
	height loss <4 cm	height loss ≥4 cm	P	height loss <4 cm	height loss ≥4 cm	P
White blood cell count (^10^9^/L)	6.03±1.39	6.32±1.41	0.127	5.44±1.26	5.63±1.33	0.203
Red blood cell count (^10^12^/L)	4.97±0.43	4.89±0.53	0.353	4.49±0.36	4.41±0.33	0.074
Hemoglobin concentration (g/L)	152.28±11.11	147.34±13.87	**0.006**	133.73±9.92	133.6±8.60	0.845
Blood platelet count (^10^9^/L)	221.05±52.25	208.98±59.76	0.068	235.97±51.77	218.71±48.90	**0.009**
Neutrophil ratio (%)	51.45±20.44	41.02±29.72	0.306	50.69±20.87	46.65±25.98	0.965
Lymphocyte ratio (%)	35.24±10.79	38.9±15.76	0.08	36.64±10.68	36.75±11.02	0.913
Eosinophil ratio (%)	6.66±10.56	12.76±14.34	**0.001**	6.58±11.54	10.35±16.11	0.449
Basophil ratio (%)	0.95±1.00	1.41±1.43	**0.002**	0.89±0.93	0.99±1.07	0.801
Fasting plasma glucose (mmol/L)	5.59±1.46	6.12±2.23	**0.045**	5.26±1.06	5.58±1.39	0.154
Glycosylated hemoglobin (%)	5.95±0.85	6.43±1.39	**<0.001**	5.85±0.68	6.29±1.42	**<0.001**
25-Hydroxyvitamin D (nmol/L)	46.82±16.36	48.61±16.75	0.373	44.82±15.52	41.73±12.46	0.102
Serum calcium (mmol/L)	2.30±0.10	2.31±0.10	0.566	2.3±0.10	2.3±0.09	0.852
Serum potassium (mmol/L)	4.07±0.31	4.09±0.40	0.398	4.05±0.31	4.11±0.31	0.21
Serum sodium (mmol/L)	140.87±1.86	141.91±1.99	**0.005**	141.02±2.34	141.62±1.88	0.218
Serum chloride (mmol/L)	103.40±2.06	103.61±1.89	0.334	104.17±2.41	104.57±1.96	0.51
Blood urea nitrogen (mmol/L)	5.40±1.23	5.56±1.56	0.496	5.01±1.22	5.59±1.39	**<0.001**
Creatinine (μmol/L)	81.77±12.88	83.45±13.74	0.398	61.78±8.94	60.86±9.44	0.354
Uric acid (μmol/L)	372.01±73.09	365.71±77.3	0.554	289.28±61.56	290.92±59.33	0.767
Alanine aminotransferase (u/L)	24.18±13.06	22.26±11.64	0.242	18.91±11.24	18.85±15.07	0.277
Aspartate aminotransferase (u/L)	22.58±7.83	21.52±6.27	0.485	21.16±7.00	22.78±10.42	0.397
Total bilirubin (umol/L)	13.31±4.79	12.74±4.02	0.314	10.56±3.52	10.69±3.34	0.727
Direct bilirubin (umol/L)	4.29±1.48	4.26±1.49	0.934	3.36±1.02	3.38±1.05	0.942
Indirect bilirubin (umol/L)	9.12±3.60	8.55±2.78	0.085	7.26±2.84	7.35±2.60	0.717
Serum total protein (g/L)	72.11±3.83	71.68±4.12	0.563	72.68±3.82	73.23±3.78	0.347
Serum albumin (g/L)	45.3±2.51	44.31±2.29	**0.002**	44.89±2.42	44.24±2.74	**0.035**
Serum globulin (g/L)	26.81±3.18	27.38±3.68	0.072	27.79±3.19	28.99±3.59	**0.008**
Total cholesterol (mmol/L)	4.98±0.95	4.86±1.07	0.309	5.25±0.88	5.26±1.01	0.896
Triglyceride (mmol/L)	1.95±1.55	1.66±0.85	0.473	1.46±0.89	1.53±0.66	0.07
HDL-C (mmol/L)	1.23±0.29	1.25±0.27	0.86	1.51±0.34	1.52±0.31	0.929
LDL-C (mmol/L)	3.17±0.82	3.14±0.96	0.765	3.29±0.83	3.32±0.92	0.822
Serum total bile acid (μmol/L)	4.81±3.73	4.59±3.23	0.576	4.6±4.08	5.29±5.92	0.706

HDL-C, high-density lipoprotein cholesterol; LDL-C, low-density lipoprotein cholesterol.

Significant differences are shown in bold.

The multivariate logistic regression analysis was performed using two models: model 1, which included clinical variables, and model 2, which incorporated biochemical variables. In men, the clinical variables significantly associated with a height loss of ≥4 cm were age (odds ratio [OR] = 1.182, 95% confidence interval [CI]: 1.111-1.258), rheumatoid arthritis (OR = 0.857, 95% CI: 0.788-0.933) and type 2 diabetes (OR = 3.012, 95%CI: 1.193-7.604). These variables were selected for the prediction model 1, which achieved an area under the receiver operating characteristic (ROC) curve of 92.6%. Among the biochemical variables analyzed in men, eosinophil ratio (OR = 1.448, 95% CI: 1.11-1.89), fasting plasma glucose (OR = 0.469, 95% CI: 0.287-0.768), glycated hemoglobin (OR = 5.65, 95% CI: 2.511-12.711), and serum sodium levels (OR = 1.328, 95% CI: 1.112-1.585) showed significant associations with a height loss of ≥4 cm, and were thus included in prediction model 2, which yield an ROC area of 87.3% (**Table 5**). In the female population, age (OR = 1.278, 95% CI: 1.182-1.382) and height (OR = 0.802, 95% CI: 0.714-0.901) in clinical variables exhibited significant correlations with a height loss of ≥4 cm, and were thus included in prediction model 1, which achieved an ROC AUC of 79%. Among biochemical variables, platelet count (OR = 0.994, 95% CI: 0.989-1.000), glycosylated hemoglobin (OR = 1.413, 95% CI: 1.090-1.832), BUN levels (OR = 1.416, 95% CI: 1.147-1.747) and serum globulin levels (OR = 1.092, 95% CI: 1.01-1.18) were significantly associated with height loss ≥4 cm and were therefore incorporated into model 2, and the area under the ROC curve of model 2 was 68.1% (**Table 6**). The Hosmer-Lemeshow test showed that the four groups of logistic models fitted well as a whole (P > 0.05).

**Table 5:** Factors associated with height loss in different groups of Chinese men.

Variable	Model 1		Model 2	
	OR (95% CI)	P	OR (95% CI)	P
Age (years)	1.182 (1.111-1.258)	<0.001		
Rheumatoid arthritis (yes vs no)	0.857 (0.788-0.933)	<0.001		
T2DM (yes vs no)	3.012 (1.193-7.604)	0.02		
Basophil ratio (%)			1.448 (1.11-1.89)	0.006
Fasting plasma glucose (mmol/L)			0.469 (0.287-0.768)	0.003
Glycosylated hemoglobin (%)			5.65 (2.511-12.711)	<0.001
Serum sodium (mmol/L)			1.328 (1.112-1.585)	0.002
	Chi-Square	p-value	Chi-Square	p-value
Hosmer-Lemeshow Test	2.852	0.943	8.95	0.347
	AUROC (95% CI)		AUROC (95% CI)	
Prediction Accuracy	0.926		0.79	
	(0.891-0.961)		(0.706-0.875)	

Model 1, included clinical variables with significant differences in univariate analysis.

Model 2, included biochemical variables with significant differences in univariate analysis.

**Table 6:** Factors associated with height loss in different groups of Chinese women.

Variable	Model 1		Model 2	
	OR (95% CI)	P	OR (95% CI)	P
Age (years)	1.278 (1.182-1.382)	<0.001		
Height (cm)	0.802 (0.714-0.901)	<0.001		
Blood platelet count (^10^9^/L)			0.994 (0.989-1.000)	0.043
Glycosylated hemoglobin (%)			1.413 (1.090-1.832)	0.009
Blood urea nitrogen (mmol/L)			1.416 (1.147-1.747)	0.001
Serum globulin (g/L)			1.092 (1.01-1.18)	0.027
	Chi-Square	p-value	Chi-Square	p-value
Hosmer-Lemeshow Test	3.505	0.899	8.244	0.410
	AUROC (95% CI)		AUROC (95% CI)	
Prediction Accuracy	0.873		0.681	
	(0.830-0.916)		(0.611-0.751)	

Model 1, included clinical variables with significant differences in univariate analysis.

Model 2, included biochemical variables with significant differences in univariate analysis.

## Discussion

This study was conducted in a systematically recruited cohort from the Health Improvement Program of Bone (HOPE). Our study identified predictive models for height loss. When only clinical risk factors were collected, height loss of ≥4 cm was predicted by age, rheumatoid arthritis, and type 2 diabetes in men, and by age and height in women. When only laboratory data were available, height loss of ≥4 cm was predicted by basophil ratio, fasting plasma glucose, glycated hemoglobin, and serum sodium in men and by platelets, glycated hemoglobin, blood urea nitrogen, and serum globulin in women. The risk factors for men and women are not exactly the same, and the exact reason for this difference between men and women is not clear. Hormone changes and health behaviors may explain this difference.

The associations of increasing age and height loss observed in this study are in line with previous findings. Mai et al. found that age was significantly associated with marked height loss (12). Fernihough and McGovern found that loss of height is higher among older age groups (25). Aging is a process closely related to the increase of hypoxia and oxidative stress (26, 27). It is reported that the increase of hypoxia and oxidative stress is related to the known causes of height decline, such as intervertebral disc disease and osteoporosis (28).

In our study, we observed that the rate of height loss accelerates after age 60, consistent with the reports of Chodzko-Zajko et al (29). Height loss is associated with the aging of bones, muscles, and joints (30). Research by Legakis et al. indicates that spinal bone density declines most rapidly around age 55 (31). Additionally, other studies have shown that muscle strength begins to decline from age 40, with this decline accelerating after the ages of 65 and 70 (29). Therefore, the acceleration of height loss after age 60 may result from the combined effects of multiple factors.

This study found that height loss was negatively correlated with height in Chinese women. Studies by Nishikura et al. have shown that height is associated with a higher risk of vertebral fractures in women, and they presume that individuals with greater height are more prone to vertebral compression fractures because of greater compressive forces applied to the lower vertebrae (32). In this case, elderly women are at a higher risk of osteoporosis and may be more likely to suffer vertebral fractures (VFs) than elderly men (32).

Our study found that rheumatoid arthritis is associated with less height loss in Chinese men. Rheumatoid arthritis (RA) itself is a risk factor for the development of osteoporosis and VFs, and this risk increases with the duration and severity of the disease (33). We believe that this may be related to the fact that some drugs for the treatment of RA, including nonsteroidal anti-inflammatory drugs (NSAIDs), disease-modifying antirheumatic drugs (DMARDs) and biological agents, have certain effects on bone metabolism disorders, in addition to glucocorticoids, which are often reported to aggravate bone metabolism disorders in RA. For example, methotrexate, currently the most widely used anti-rheumatic drug to manage the condition, cannot significantly change the bone mineral density of the femoral neck and lumbar spine in RA patients at therapeutic doses, and can reduce the activity of osteoclasts and fibroblastic cells and hinder bone absorption (34–36).

This study found that in Chinese men, a history of type 2 diabetes and glycated hemoglobin were positively correlated with height loss; in Chinese women, although the correlation of a history of type 2 diabetes disappeared after correction for other clinical risk factors, glycated hemoglobin remained positively correlated with height loss after adjusting for other related hematological indices. Previous studies have shown that diabetes is an established risk factor both for intervertebral disc degeneration (37) and osteoporosis-related fractures (38), which are major risk factors for height loss among adults. Shimizu et al. also found that there is a positive correlation between glycosylated hemoglobin (HbA1c) and height loss among Japanese workers, even if HbA1c is within the normal range, higher HbA1c is an important risk factor for height loss among workers (39). They believe that although the mechanism underlying the positive association between HbA1c and height loss is currently unknown, since height loss is positively associated with all-cause and cardiovascular mortality, vascular endothelial status might underlie this association (39). Studies indicate that the beneficial effects of maintaining the endothelium could prevent height loss. However, how the status of the endothelium influences vertebral fractures and decreases in intervertebral disc height, which are major causes of height loss among adults, remains unknown (39).

Our research showed that high fasting plasma glucose (FPG) was a protective factor for height loss. The study by Sung et al. found that the risk of osteoporosis in Koreans decreased with the increase in fasting plasma glucose and compared to subjects with normal fasting plasma glucose, subjects with impaired fasting glucose (IFG) and diabetes had lower risk of osteoporosis. These results show that the increase in fasting plasma glucose in Koreans has a protective effect on osteoporosis (40). Similarly, Lu et al.’s research shows that low FPG may increase the risk of osteoporosis in non-diabetic elderly women in China (41). Iki et al. also found that hyperglycemia is associated with an increased risk of fracture among elderly men in Japanese communities (42), which was not observed in our study. The relationship between FPG and osteoporosis is complex. Mendel’s randomized study clarified the relationship between FPG and BMD at the genetic level, and *in vivo* experiments showed that high glucose levels might interfere with osteoclast differentiation and inhibit osteoclast-mediated bone matrix degradation, thus leading to an increase in BMD (41). In conclusion, healthy women over 40 years old in China need to further investigate whether there is significant height loss when they encounter low FPG, so as to identify bone diseases early.

In our study, the higher the serum sodium concentration, the greater the likelihood of male height loss of ≥4 cm. Wu et al.’s research shows that hypernatremia can induce the formation of human bone-resorbing osteoclasts, and the differentiation and function of mouse and human osteoclasts are significantly increased at a moderate NaCl concentration (43). Studies also show that mild to moderate chronic hyponatremia is a risk factor for osteoporosis and fractures (44).

In our study, the higher the platelet count, the lower the likelihood that women lose more than 4 cm in height. Previous studies have shown that platelets (PLT), cytoplasmic fragments from bone marrow megakaryocytes, play a key role in bone homeostasis and can regulate bone formation and resorption, but the exact mechanism has not yet been proposed. In fact, it has been proven that the plasma platelet-derived growth factor-BB (PDGF-BB) levels of normal young women are maintained by estrogen and play a major role in postmenopausal osteoporosis (45).

Serum globulin is used as a sign of inflammation in clinical practice (46), and inflammation is a known factor of intervertebral disc degeneration (47) and osteoporosis (48), so it may also be the basis of the positive correlation between serum globulin and height decline. In addition, studies have shown that serum osteocalcin (OC) level, which is specifically expressed in osteoblast lineage cells, has been found to be correlated with basophil count (49). At present, the relationship between basophil ratio, blood urea nitrogen and serum globulin and bone metabolism is not clear, and further research is needed.

Our findings have implications for fracture risk assessment of healthy people over 40 years of age in China. Diagnostic methods, including bone densitometry and bone X-ray photography, are not always available and increase the total cost of the health care system (50). Height loss is a simple, safe and low-cost measurement method, and clinical guidelines often suggest that a series of measurements of height should be taken as part of routine fracture risk assessment (22, 23). However, health care providers still often use self-reported height instead of actually measured height recorded in charts, which may lead to unrecognized height decline (51). Therefore, our research is useful in estimating the risk of height loss. In addition, height loss has been reported to be associated with an increased risk of fractures and mortality (12). In view of this, our findings may also help practitioners target individuals at risk of later fracture and mortality earlier.

This study reports for the first time the age-related trend of height loss and the risk factors for height loss in healthy men and women aged 40 and above in China. Our research has several limitations. First of all, the cohort mainly consists of individuals undergoing physical examinations at the Health Management Center of the Second Xiangya Hospital, so the results may not be generalizable to the wider population. Second, the evaluation of height loss was only based on the self-reported peak height. Although measuring the actual height loss would be ideal, the self-reported height loss was also valuable and comparable with actually measured height loss (52).

## Conclusion

In conclusion, our research shows that in the population of China, regardless of gender, the rate of height loss is significantly accelerated after age 60. In men, age, rheumatoid arthritis, type 2 diabetes, basophil ratio, fasting plasma glucose, glycosylated hemoglobin and blood sodium are related to height loss of ≥4 cm, while in women, age, height, platelets, glycosylated hemoglobin, blood urea nitrogen and serum globulin are related to height loss of ≥4 cm. These results help clarify the mechanisms of adult height loss. Furthermore, this prediction model may be useful not only in identifying the population at risk of height decline in China, where intervention could be beneficial, but also that early identification and treatment of these groups may have potential downstream effects on the consequences of height decline, including fractures and mortality.

## Data Availability

The original contributions presented in the study are included in the article/supplementary material. Further inquiries can be directed to the corresponding author/s.
